# NK cell function and receptor diversity in the context of HCV infection

**DOI:** 10.3389/fmicb.2015.01061

**Published:** 2015-09-30

**Authors:** Clair M. Gardiner

**Affiliations:** NK Cell Group, School of Biochemistry and Immunology, Trinity Biomedical Sciences Institute, Trinity College DublinDublin, Ireland

**Keywords:** NK cells, HCV, hepatitis C, KIR, chronic viral infection

## Abstract

Hepatitis C virus (HCV) infects over 170 million people in the world. While a minority of individuals are able to naturally clear this hepatotropic virus using their immune system, most people go on to develop a lifetime chronic infection that can result in severe liver pathology, potentially leading to liver cirrhosis and hepatic cellular carcinoma. Investigations into acute immune responses and spontaneous clearance of the virus are severely hampered by difficulties in identification of relevant patient cohorts. While the role for the adaptive immune response in viral clearance is well established, it is becoming clear that the innate immune system also impacts on HCV outcome. The innate immune response to infection is likely to influence the type of adaptive immune response that develops and will ultimately influence if the virus is cleared or develops into a chronic infection. Natural Killer (NK) cells are lymphocytes that have important anti-viral functions including direct cytotoxicity of infected cells and the production of inflammatory cytokines, e.g., IFN-γ. They are generally considered to be cells of the innate immune system, although there is increasing evidence that NK cells adapt and persist in response to particular viral infections. NK cells are altered in patients with acute and chronic HCV infection. There is increasing evidence from both cellular and genetic studies that NK cells modulate HCV outcome. This review will describe and discuss the current experimental and clinical evidence of a role for NK cells in HCV infection and describe recent discoveries that are likely to play a role in future research.

## Introduction

Hepatitis C virus (HCV) infects approximately 170 million people in the world (Alter and Seeff, 2000). HCV infects hepatocytes and while virus can be cleared by the immune system in a minority of individuals, most people develop a chronic lifetime infection associated with progressive liver disease (Shepard et al., 2005; Micallef et al., 2006; Rehermann, 2009). It is not yet clear why some individuals can spontaneously clear infection while others cannot, and both viral and host factors are likely to impact on this. Most research has focussed on the development of adaptive immunity in this context (Cooper et al., 1999; Lechner et al., 2000; Thimme et al., 2002). However, there is substantial evidence emerging to suggest that the innate immune system also significantly contributes to HCV outcome (Ge et al., 2009; Rehermann, 2009; Suppiah et al., 2009; Tanaka et al., 2009; Nattermann, 2011; Prokunina-Olsson et al., 2013). In addition to cells of the innate immune system, including Natural Killer (NK) cells, the focus on this review, cytokines such as the type 3 IFN family strongly impact on HCV outcome (Thomas et al., 2009; Tillmann et al., 2010; Dring et al., 2011), although the mechanisms involved remain elusive.

The treatment for HCV has historically been a combination of IFNα and ribavirin but this is changing with the advent of direct acting antivirals (DAAs) (Zeuzem et al., 2011, 2014; Afdhal et al., 2014). DAAs have revolutionized treatment of HCV and IFNα free therapy is currently being introduced. While further refinements of these drugs are likely, the DAAs are not without their problems. Currently, their cost is hugely expensive although this should come down in the future. At present, DAA therapy is only a viable option for developed countries. Furthermore, identification of DAA resistant variants of HCV have already been identified (Carganico et al., 2014; Hedskog et al., 2015; Ji et al., 2015). There are also likely to be complications in terms of drug interactions in particular patient subsets, e.g., patients co-infected with HIV-1 (Burgess et al., 2015; Soriano et al., 2015) and reinfection is likely to occur in high risk patients (Baumert et al., 2014). Therefore, alternative therapeutic approaches, including development of a prophylactic vaccine, remain important goals and understanding the immune response during HCV infection is key to ensure appropriate development of protective immunity (Baumert et al., 2014). Modulation of innate immunity, including NK cells, provides one potential mechanism for improving immunity to vaccination.

NK cells are lymphocytes that have traditionally been classified by Immunologists as part of the innate immune response as they can mediate rapid effector responses and do not necessarily need prior sensitization for effector functions such as cytotoxicity (Caligiuri, 2008). This view point is changing rapidly however with the demonstration of long lived, activated NK cells that have sustained functions beyond the classical “innate” time-frame (Cooper et al., 2009; Sun et al., 2009; Björkström et al., 2011). NK cells are best known for their anti-viral activities and anti-tumor activities. They can kill virally infected cells and produce IFNγ cytokine that has direct anti-viral functions in addition to modulating the adaptive immune response (Caligiuri, 2008; Wang et al., 2008). NK cells have been shown to kill HCV infected hepatocytes (Larkin et al., 2006; Stegmann et al., 2010). Indeed, there is substantial evidence accumulating to support that they play an important role in HCV infection and although they may be important during the immune response to acute infection, they may continue to play significant roles during chronic HCV infection and during treatment. Indeed, it is possible that NK cells, rather than being just a friendly force, may contribute to development of chronic infection or indeed immune mediated pathology associated with chronic infection.

This review summarizes the current studies, both genetic and cellular, that provide evidence of a role for NK cells during acute and chronic HCV infection. In addition, significant advances have been made in recent years in terms of understanding NK cell biology including an appreciation for the impact of cytomegalovirus (CMV) on NK cell responses, the discovery of tissue resident NK (trNK) cells and a growing awareness that NK cells may play previously unanticipated roles during the adaptive immune response, possibly even contributing to disease pathology. These all remain to be explored in terms of their impact during HCV infection and will be discussed in more detail later.

Hepatitis C is a human disease. HCV does not infect rodents and although progress has been made, no robust small animal models for the study of the immune response to HCV exist (Vercauteren et al., 2014). *In vitro* culture systems for HCV have been developed and while they are not very physiological, they allow dissection of particular aspects of HCV infection (Lohmann and Bartenschlager, 2014). Therefore, most studies on the role of the immune system in HCV use human cohorts and samples for analysis. HCV infects hepatocytes and it is likely that most relevant immunology occurs locally in the liver. In the case of NK cells, this is probably particularly pertinent as NK cells are particularly enriched in the liver accounting for over 30% of lymphocytes compared to a frequency of approximately 10% of peripheral blood lymphocytes in humans (Hata et al., 1990; Satoh et al., 1996; Norris et al., 1999; Doherty and O'Farrelly, 2000). While liver samples from patients with chronic HCV are relatively easier to come by, it is substantially more difficult to get liver samples from healthy controls, and our knowledge of events in the liver is relatively poor compared to information on systemic immune events during HCV infection. The limited data available suggest that differences exist between matched peripheral blood and hepatic NK cells in terms of phenotype and function, and that differences are also seen between hepatic NK cells of patients with chronic HCV compared with controls (Kawarabayashi et al., 2000; Varchetta et al., 2012). Despite this caveat, there are clear changes in systemic immune cells during infection and there is some evidence that changes observed in the periphery are similar to those seen in liver albeit with relatively lower levels of magnitude (Ahlenstiel et al., 2010).

## Genetic analysis of KIR genes provides evidence of a role for NK cells role in HCV

Evidence of a role for NK cells in HCV comes from several different sources, including genetic and cellular settings. Identifying the contribution of the immune system, including NK cells, to either resolution of infection or the development of chronic HCV infection is not a trivial task given the difficulties in identifying appropriate control cohorts. Many individuals that spontaneously resolve HCV infection are often not aware of their infection and identification of such individuals is extremely difficult (Micallef et al., 2006; Cox et al., 2009). Approaches for comparison of spontaneous resolution vs. development of chronic infection have therefore included retrospective genetic analysis of iatrogenic cohorts of patients given HCV contaminated blood products and prospective analysis of high risk patient groups, e.g., intra-venous drug users (IVDU). Other studies have used a variety of control groups, e.g., healthy normal donors or non-infected IVDU patients to compare to chronic infection but this analysis is confounded by the fact that within the control group, some of the individuals would resolve and others would develop chronic infection if infected with HCV. Heterogeneity of cohorts including ethnicity, genotype of virus, route of infection, and presence of other co-morbidities all complicate analysis as they can contribute to HCV outcome (Thimme et al., 2002; Shepard et al., 2005). Thus, each study must be examined on its own merits in terms of appropriate controls and samples numbers.

**Figure 1:**
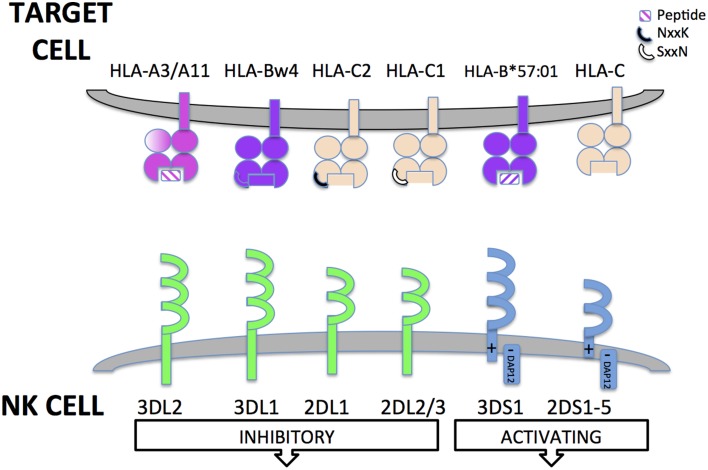
**Killer cell immunoglobulin-like receptors (KIR)**. Killer cell immunoglobulin-like receptors (KIR) are encoded for by genes found in the leukocyte receptor complex on chromosome 19. KIR have either 2 or 3 Ig extracellular domains. KIR can have either long or short cytoplasmic tails. The long cytoplasmic tails contain immune-tyrosine based inhibitory motifs (ITIMs) that transduce inhibitory signals to the NK cell upon recognition of HLA class I ligand. KIR with short cytoplasmic tails lack intrinsic signaling capabilities. However, they have a charged residue in their transmembrane domain that allows interaction with an adaptor protein (DAP12) that transduces activating signals to NK cells through Immunotyrosine based activatory motifs (ITAMs). Some HLA class I ligands are recognized by activating KIR but for most short-tailed KIR, the ligands are unknown. KIR recognize conserved epitopes of HLA class I receptors. KIR3DL2 recognize specific HLA-A alleles (HLA-A3/A11) and are sensitive to the peptide present in the antigen binding groove. KIR3DL1 recognizes the HLA-Bw4 serological epitope present in some HLA-B and HLA-A alleles (encoded by residues 77–83 of α1 domain of the HLA class I heavy chain). KIR2DL1 and KIR2DL2/3 recognize HLA-C allotypes. Specificity is determined by a dimorphism at 77xx80 of the HLA class I heavy chain. KIR2DL1 recognizes HLA-C2 epitope while KIR2DL2/3 recognize the HLA-C1 epitope. KIR2DS1 recognizes HLA-C2 and KIR2DS2 recognizes HLA-C1; 2DS4; KIR3DS1 has recently been described to recognize B^*^57:01 in a peptide specific manner. Some non-HLA encoded ligands for activating KIR have been identified, e.g., viral haemagglutinin but for many, no physiological ligands have yet been defined.

One of the biggest breakthroughs in NK cell biology was the discovery of a family of germ line encoded receptors that are expressed almost exclusively by NK cells and that are key to NK cell recognition and function (Vilches and Parham, 2002). Thirteen highly polymorphic Killer cell immunoglobulin-like receptors (KIR) genes reside on chromosome 19 in the Leukocyte Receptor Complex. Some of the genes encode inhibitory receptors and other appear to encode activating receptors, although their biology is less well understood (see Figure 1). KIR receptors recognize conserved epitopes on HLA class I molecules. In its simplest form, a dimorphism at residues 77xx80 of the HLA-C heavy chain encodes either HLA-C1 or HLA-C2 epitopes that are recognized by KIR2DL3 (including KIR2DL2 variants) and KIR2DL1 respectively (Vilches and Parham, 2002). Furthermore, this same region of the HLA-B heavy chain encodes the Bw4 epitope that provides a ligand for KIR3DL1 receptor. Thus, HLA-C1, HLA-C2, and HLA-Bw4 are the three HLA class I ligands that are recognized by KIR. However, KIR recognition of HLA is more complex than this as KIR2DL2/3 receptors have some cross-reactivity with HLA-C2 alleles (Moesta et al., 2008). Furthermore, although it will not be discussed in this review, there is also evidence that KIR recognition is influenced by peptide presented by HLA class I molecules and that this can alter NK cell recognition and function (Rajagopalan and Long, 1997; Cassidy et al., 2014).

Our knowledge of basic KIR biology (expression, specificity, function) lags well behind the advances we have made in terms of KIR genetics. This is partly due to the difficulties in developing specific reagents for proteins with a high degree of similarity (Gardiner, 2008). However, the number of KIR genes, the high degree of functional polymorphism of these genes, e.g., impact of KIR3DL1 allelic variation on HLA-Bw4 recognition (O'Connor et al., 2007), the existence of complimentary receptor systems (CD94/NKG2), the polygenic and polymorphic nature of HLA-class I ligands and the lack of KIR in the mouse make functional cell biology experiments challenging.

Despite this, analysis of KIR genes is relatively straightforward although the number of KIR genes, and the common requirement to also study HLA class I (given the epistatic nature of functional interactions), mean that large cohorts are required for studies to have adequate statistical power. In terms of HCV research, many studies have investigated a role for KIR genes and their ligands in infection outcome or treatment response. In terms of evidence that NK cell genes contribute to either spontaneous resolution or chronic infection, particular combinations of KIR and HLA have been identified that impact HCV infection outcome. One of the first large studies identified that *KIR2DL3*, when present on a homozygous ligand background (*HLA-C1/C1*), was associated with spontaneous resolution of HCV infection (Khakoo et al., 2004). This cohort was very heterogeneous but had large sample numbers (*n* = 1037). The authors hypothesized that as KIR2DL3 binds HLA-C1 with a weaker affinity (compared with KIR2DL2 binding of HLA-C1), it would be easier to overcome this inhibitory interaction; therefore, NK cells in individuals with this combination of receptor and ligand would be more easily activated during HCV infection resulting in a better outcome. When stratified into patients infected by transfusion (predicted higher dose of HCV) or non-transfusion (e.g., needle-stick injuries with predicted lower dose of HCV exposure), the beneficial effect of *KIR2DL3/HLA-C1/C1* was only seen with the non-transfusion group, suggesting that NK cells may make a more effective contribution to the immune response when there is a more limited exposure to virus. The relevance of this *KIR2DL3/HLA-C1/C1* genotype has been supported as beneficial in acute HCV infection by the same group (Knapp et al., 2010) and a higher frequency of 2DL3 expressing NK cells has been observed in a cohort of exposed uninfected individuals (Thoens et al., 2014); however, other studies, albeit with different cohorts, have not observed this association (Montes-Cano et al., 2005; Rauch et al., 2007; Thoens et al., 2014).

We have previously published on the contribution of KIR genes to HCV outcome in the Irish anti-D cohort (Dring et al., 2011). These patients were infected by HCV contaminated anti-D immunoglobulin in 1977/79. As the cohort were infected from a single source, they provide a rare opportunity to look at the impact of the host immune response in HCV outcome in the absence of confounding variables, e.g., virus heterogeneity. In this group, we found a trend toward *KIR2DL3/HLA-C1* contributing to resolution of HCV infection as the frequency of *KIR2DL3*+*/HLA-C1/-C1* was higher in patients that resolved (*n* = 247) compared to those that developed chronic infection (*n* = 296). However, the Khakoo model predicts that this effect should be stronger on a *KIR/HLA* homozygous background but we did not find this in our cohort (Khakoo et al., 2004). One possible explanation for this difference might be due to differences in the route of viral transmission. Our cohort is more similar to the patients that were infected through blood transfusion products for whom no beneficial effect of *KIR2DL3/HLA-C1* was observed. Although our data did not reach statistical significance, they suggested that NK cells could potentially contribute to resolution of HCV infection through *KIR2DL3/HLA-C1*, even when patients are initially exposed to higher levels of HCV.

We also identified KIR B haplotype associated gene, *KIR2DS3*, as a risk factor in the Irish anti-D cohort for development of chronic HCV infection when present with *HLA-C2* (Dring et al., 2011). This relevance of this finding was subsequently confirmed when it was shown to also be associated with HCV treatment failure in an Irish cohort of HCV/HIV-1 co-infected patients (Keane et al., 2013). We do not yet understand the mechanism for the association observed as *KIR2DS3* is not expressed at the cell surface (VandenBussche et al., 2009) and there is no ligand identified for KIR2DS3 (Moesta et al., 2010). However, the *HLA*-*C2* association makes it likely that a KIR gene in linkage disequilibrium with *KIR2DS3* could be responsible for the biological effect. Given that *KIR2DS3* was found in a haplotype with *KIR2DL1* and *KIR2DL2* and these can both bind HLA-C2, they are good candidate genes for the phenotype observed. Another important point is that gene level analysis of KIR is only informative up to a point. In our cohort, *KIR2DL1* was present in almost all donors and therefore was not informative as a marker of clinical outcome. We know that KIR allelic variation can affect NK cell function and it is likely that particular allotypes are responsible for particular phenotypes. Given that we saw no association with outcome for *KIR2DL2* in our study, it is more likely that a particular allele of *KIR2DL1*, in combination with its *HLA-C2* encoded ligand, may be detrimental in HCV in our cohort.

It is also worth remembering that phenotypes are a result of the net sum of interactions between KIR and HLA and that allele level haplotypes rather than individual genes may be more relevant in these situations. Also, the KIR/HLA system is only one of the receptor ligand interactions that modulate NK cell function and thus, effects observed are likely to be subtle. Finally, there is a lot of genetic variability within KIR genes and haplotypes and between different ethnic and geographical populations. For example, there are two main KIR haplotypes described in human populations. The KIR A haplotype is characterized by predominantly inhibitory KIR that bind HLA class I ligands while the KIR B haplotype contains more diverse genes including short-tailed KIR that are potentially encode activating receptors and for many of whom, their ligands remain undefined. Populations vary in the relatively frequencies of these haplotypes; the Japanese and Australian aboriginals are at extremes with high KIR A and B haplotypes respectively (Gonzalez-Galarza et al., 2011; Parham et al., 2012). It is likely that genetic associations with disease discovered may not be relevant in all global populations. However, such genetic studies are essential for simplifying some of the complexities and defining experiments to test the molecular basis for the observations.

## NK cells are activated in acute HCV but this does not appear to correlate with outcome

Genetics studies, although informative, are generally limited to retrospective analysis and do not provide insights into the immune events that occur immediately after infection and that ultimately affect outcome. However, prospective studies on acute HCV are hampered due to difficulties identifying patients that have acute HCV infection and these studies generally have low patient numbers. This caveat also precludes analysis of multigenic complex gene families such as the KIR, and prospective studies mainly focus on NK cell subsets and expression of key activating and inhibiting receptors. Unfortunately, conflicting reports abound with respect to almost all aspects of NK cell function and phenotype during acute HCV infection and general conclusions discussed below must be considered in this context.

In general, there is agreement that the frequency of NK cells present does not correlate with resolution of infection or development of chronic HCV infection (Alter et al., 2011; Golden-Mason et al., 2014; Kokordelis et al., 2014). There is also general agreement that there is a relative expansion of CD56^bright^ subset of NK cells during acute HCV infection with a concomitant decrease observed in CD56^dim^ subset of cells (Morishima et al., 2006; Golden-Mason et al., 2008; Alter et al., 2011). These subsets are characterized by different functions. The CD56^bright^ NK cells (KIR negative) are potent producers of IFNγ and less cytotoxic than their CD56^dim^ counterparts (Baume et al., 1992; Fehniger et al., 1999; Jacobs et al., 2001). Some reports find that NK cells are activated during acute HCV infection as evidenced by changes in NK cell phenotype including expression of activation receptors, e.g., NKG2D (Amadei et al., 2010; Kokordelis et al., 2014) although expression of other receptors associated with NK cell activation, e.g., NKp44 do not change (Pelletier et al., 2010; Alter et al., 2011; Golden-Mason et al., 2014). Most reports suggest that cytotoxic functions (TRAIL and CD107a degranulation assays) of NK cells are increased during acute infection (Amadei et al., 2010; Pelletier et al., 2010; Alter et al., 2011; Werner et al., 2013; Golden-Mason et al., 2014). However, there is no evidence to suggest that NK cell killing potential during acute infection directly affects HCV outcome (Amadei et al., 2010). Similarly, although the data are again conflicted (Pelletier et al., 2010), there is a general consensus that NK cells are activated to produce more IFNγ during acute infection (Amadei et al., 2010; Werner et al., 2013; Kokordelis et al., 2014), but this does not appear to impact on disease outcome (Amadei et al., 2010).

The reality is that we do not yet understand the role that NK cells play in the early immune response to HCV and not even which effector functions are likely to be more important. Some evidence suggests that IFN-γ might be key in inhibiting viral replication (Thimme et al., 2002; Wang et al., 2008; Kokordelis et al., 2014) while other data, showing that NK cell degranulation potential during acute infection correlates with development of a robust CTL response, supports NK cell cytotoxic functions as a more important effector mechanism (Pelletier et al., 2010; Werner et al., 2013). In addition to their roles as effector cells, NK cells also modulate the development of adaptive immunity. This is important for our understanding of how an adaptive immune response develops but also in terms of potentially targeting NK cells during HCV vaccination to improve protective immunity generated. More basic research defining the role of NK cell effector and regulatory functions in the immune response to HCV is required (Golden-Mason et al., 2014).

## NK cells have altered phenotype and function in chronic HCV infection

Most studies on the role of NK cells in HCV infection have focussed on chronic infection (as patient samples are readily available) and thus we have more data available about this aspect of infection compared to any other. It is worth remembering that by the time chronic infection is established, the immune system has had an opportunity to clear the virus and has failed to do so. By studying the immune systems of patients with chronic infection, we aim to understand aspects of the immune system that contribute to the development of chronic infection. However, the nature of a chronic infection means that there will be continued production of HCV virus particles over the lifetime of the host. Thus, the environment in which the immune system finds itself will be different. Prolonged presence of the virus can impact of the immune system, e.g., induction of immune evasion mechanisms (Herzer et al., 2003; Séne et al., 2010; Park and Rehermann, 2014) but also, continuous and chronic activation of immune cells can lead to altered functions, including their becoming “exhausted” or anergic (Park and Rehermann, 2014). Thus, it can be difficult to dissect cause vs. effect, e.g., does an altered immune function in patients predispose to the development of chronic infection or is it caused as a result of chronic virus infection? Furthermore, it is likely that dysregulated immune activities can also contribute to later onset pathology associated with chronic viral infection, e.g., fibrosis. Therefore, analysis of NK cells during chronic infection needs to be considered against this background.

There is consistency across a number of studies to suggest that although the overall frequency of NK cells is either unchanged (or low) during chronic infection (Morishima et al., 2006; Golden-Mason et al., 2008; Alter et al., 2011), there is a relative dysregulation of NK cell subsets, with a decrease in the CD56^dim^ cells and conversely, an increase in the frequency of CD56^bright^ cells in the peripheral blood of patients compared to healthy normal controls (Morishima et al., 2006; Golden-Mason et al., 2008; Alter et al., 2011). Although these NK cell subsets are ascribed general, albeit simplistic, functional activities, e.g., CD56^bright^ cells are generally considered more potent at producing IFNγ, in reality, the functions of these subsets become more blurred. For example, as CD56^dim^ cells account for approximately 90% of NK cells, they can be a relatively major source of IFNγ when activated (Anfossi et al., 2006; Horowitz et al., 2010). Indeed, there is evidence to support that CD56^dim^ cells can be a potent source of rapid IFNγ production, prior to production by CD56^bright^ cells (De Maria et al., 2011).

In terms of alterations in receptor expression, there are few consistent findings. Expression of natural cytotoxicity receptors (NCR; NKp30, NKp44, and NKp46) or NKG2D, as activating receptors, are reported as either up, down or not changing on peripheral blood NK cells depending on the study (De Maria et al., 2007; Ahlenstiel et al., 2010; Pelletier et al., 2010; Alter et al., 2011). There is so much heterogeneity in terms of cohorts in terms of both patient characteristics and viral factors that it is difficult to find studies that are directly comparable and which validate findings in a particular context.

One receptor that appears to be consistently upregulated in patients with chronic HCV is NKG2A (Jinushi et al., 2004; Alter et al., 2011; Golden-Mason et al., 2014). NKG2A forms a heterodimer with CD94 on NK cells. It recognizes HLA-E molecules on target cells and inhibits NK cell function (Braud et al., 1998; Lee et al., 1998). During chronic HCV infection, intrahepatic HLA-E expression is increased. This is due in part to binding of a HCV derived peptide that stabilizes expression at the cell surface; this also facilitates inhibition of NK cell function (Nattermann et al., 2005).

Given that cytotoxicity and cytokine production are two key functions that are important in the NK cell response to viral infection, these are been assessed in many studies. Along with the heterogeneity of cohorts mentioned above, there is also variability in the literature in terms of how experiments are done, controls groups used, subsets of cells are analyzed, how analysis is performed, e.g., frequency of expression (%) vs. level of expression (mfi) and how the data are presented. While there are occasional reports of unchanged (De Maria et al., 2007) or even higher production (Golden-Mason et al., 2008), there is a growing consensus, across a range of experimental platforms, that peripheral blood NK cells from patients with chronic HCV infection make less IFNγ than healthy normal individuals (Jinushi et al., 2004; Oliviero et al., 2009; Ahlenstiel et al., 2010; Alter et al., 2011).

The cytotoxic functions of NK cells appear to be transiently increased during acute HCV viral infection (Amadei et al., 2010; Pelletier et al., 2010; Werner et al., 2013). However, many studies have confirmed that NK cell cytotoxicity is not impaired during chronic HCV infection (Morishima et al., 2006; Golden-Mason et al., 2008; Ahlenstiel et al., 2010; Alter et al., 2011). These general observations are supported by one particular study that stratified patients with chronic HCV on the basis of ALT levels (Ahlenstiel et al., 2010). Patients with normal ALT levels had normal NK cell activity while those with higher ALT levels, associated with active infection, had higher cytotoxicity against target cells. Overall, these data suggest that if NK cell cytotoxic functions are important for viral clearance early after infection, they are no longer an effective mechanism for viral clearance once infection has become established. One consideration with most of the cell function studies cited is that non-hepatic target cells have generally been used and occasionally, differences have been observed in terms of NK cell responses between common haematopoietic target cells, e.g., K562, 721.221 and hepatic target cells (Jinushi et al., 2004). Confirmation of these findings by several laboratories, e.g., using the HCV replicon system would be a worthy endeavor.

Upon recognition of a target cell, NK cells primarily kill using molecules contained in their cytotoxic granules (measured in the CD107a assay). However, they can also kill by receptor mediated ligation using TRAIL, fas-ligand and membrane bound lymphotoxin, to trigger apoptosis in target cells. Of these, TRAIL expression has been reported to play a role in NK cell recognition of HCV infected cells (Ahlenstiel et al., 2010; Stegmann et al., 2010). In general, a modest upregulation of TRAIL is reported during acute infection (Werner et al., 2013; Golden-Mason et al., 2014) and it appears to remain elevated during chronic infection. TRAIL receptors are also upregulated on HCV infected hepatocytes (Jang et al., 2014). Increased TRAIL expression on NK cells has also been associated with an ability to induce apoptosis of hepatitis stellate cells and potentially inhibit HCV associated development of fibrosis (Glässner et al., 2012). However, it has also been reported that TRAIL is decreased on hepatic NK cells of HCV infected individuals compared to controls (Varchetta et al., 2012). Thus, it is not yet clear the role that TRAIL plays in the NK cell immune response during HCV infection.

In addition to these direct effector functions, NK cells can regulate the development of adaptive immunity by a variety of mechanisms, including interacting with Dendritic cells (DC). There is a reciprocal interaction between NK cells and DC, and activated NK cells have the potential to kill immature Dendritic Cells (iDC), thereby limiting the strength of the downstream adaptive immune response (Ferlazzo and Moretta, 2014). It has been shown in a co-culture system with hepatic cells, that NK cells from patients with chronic HCV infection had a reduced ability to activate DC compared with NK cells from healthy normal donors. NK cells from the patients also had higher production of immunosuppressive cytokines, TGFβ and IL10 (Jinushi et al., 2004). Thus, as a result of altered NK cells, DCs from patients with chronic HCV are less likely to drive a strong adaptive immune response.

## Variation in control samples hinders interpretation of hepatic NK cell function during chronic HCV infection

Almost all the data discussed in this review relates to peripheral blood samples from patients with HCV infection. There is only limited data from liver samples from patients with chronic HCV. These studies suggest that hepatic NK cell numbers are either decreased (Kawarabayashi et al., 2000; Boisvert et al., 2003; Bonorino et al., 2009) or unchanged (Deignan et al., 2002) in patients with chronic HCV. Hepatic NK cells in patients with chronic HCV may have lower IFNγ production (Kawarabayashi et al., 2000) and cytotoxicity/degranulation capacity (Kawarabayashi et al., 2000; Varchetta et al., 2012) compared to controls. Furthermore, two studies have shown an association between high cytotoxicity and reduced liver fibrosis during chronic HCV (Morishima et al., 2006; Fugier et al., 2014). However, it should be pointed out that relatively poor cell yields limit experiments possible while sampling times of liver with respect to disease (e.g., non-cirrhotic vs. cirrhotic liver) can also affect results. Furthermore, there is no consistency amongst the control groups used in the various studies which makes direct comparisons difficult. Some studies have used “normal” liver from cancer patients (with cancers other than hepatic cellular carcinoma, Kawarabayashi et al., 2000) but these are not ideal as NK cells can be affected in cancer patients and some of the livers sampled had metastatic disease of the liver (Kawarabayashi et al., 2000). Other studies compared paired liver and blood samples but had no healthy liver to provide context for the liver data (Ahlenstiel et al., 2010, 2011). Yet other studies have used Non-Alcoholic Fatty Liver Disease (NAFLD) liver biopsy samples (Fugier et al., 2014), donor liver samples (Deignan et al., 2002) or liver biopsies taking during non-liver related procedures (Varchetta et al., 2012) as controls samples. Appropriate control groups and adequate sample size of cohorts remain significant hurdles to this important analysis.

### NK cells in treatment

The treatment for HCV is rapidly changing with the introduction of DAAs. However, for many years IFNα and ribavirin was the standard of care therapy for HCV infection. IFNα is a type 1 IFN that can potently activate NK cells. In light of this, many studies were performed looking at the effect of IFNα on NK cells during treatment and correlating these with treatment success or failure. Given that IFNα is likely to phased out of clinical use as a front line therapy for HCV and that there is no particular rationale for DAAs impacting on the NK cell responses other than to normalize immune functions (Serti et al., 2015), such treatment studies are unlikely to be a focus on HCV research into the future.

## The changing landscape of NK cell biology: Implications for future HCV research

Our knowledge of NK cells is expanding exponentially. Over the last 15 years, the discovery of complex receptor systems, complex education processes (Fernandez et al., 2005; Kim et al., 2005; Anfossi et al., 2006; Joncker et al., 2009; Elliott and Yokoyama, 2011) and “memory” like functions of NK cells have changed the way we view NK cells. No longer considered simple cells of the innate immune system, NK cells are now recognized to play roles beyond what we previously anticipated or expected. All of these discoveries are likely to impact our understanding of the role that NK cells play in HCV infection.

There are some relatively newer concepts that may prove to be particularly important in HCV infection. Firstly, there is strong evidence emerging that activated NK cells may not always be beneficial during the immune response to virus. Data from the LCMV mouse model shows outcome is sensitive to the relative frequencies of NK cells and T lymphocytes (Waggoner et al., 2012). In particular, NK cells regulated the adaptive immune response, by killing CD4+ lymphocytes, and the system could be manipulated to demonstrate that NK cells contributed to immune mediated pathology that was lethal for the animals (Waggoner et al., 2012). Furthermore, NK cells can potentially impact on the development of immune memory. In NKp46 deficient mice, an early hyper-responsive NK cell response was associated with impaired development of specific memory responses (Narni-Mancinelli et al., 2012). Similarly, NK cell depletion can lead to higher CD8+ T cells present with enhanced memory responses (Soderquest et al., 2011). More recently, data has emerged suggesting that this may also be the case for human NK cells as they were shown to kill CD8+ T cells during chronic Hepatitis B virus (HBV) infection and regulate their functions (Peppa et al., 2013). These various data challenge the paradigm that NK cells are always beneficial in the anti-viral immune response and serve to illustrate how little we actually know about how NK cells modulate the development of specific adaptive immunity. Importantly, they also support the exciting concept that by modulating NK cell responses during vaccination, we could promote the development of enhanced protective immunity to HCV vaccines, a major goal of the HCV field.

There is also data emerging that supports the presence of tissue specific NK cells and this may have consequences for organ specific disease such as HCV infection. The discovery of NK-like cells (originally termed NK22, now reclassified as ILC3) in mucosal tissues highlighted the potential role for NK-like cells in specific organs (Colonna, 2009). While ILC3 have now been shown to be from a separate lineage, they have many features in common with NK cells (Artis and Spits, 2015). More compelling however, is the demonstration of specific tissue resident NK (trNK) cells that appear to arise from separate lineages (Cortez et al., 2014; Sojka et al., 2014). Parabiotic mice clearly showed that NK cells resident in the liver remained there while splenic NK cells freely circulated between the host and the parabiont (Peng et al., 2013). Further work has shown that murine liver, uterus and skin all have particular NK cell subsets characterized by particular phenotypes, transcriptomes and transcription factors (Sojka et al., 2014). It is likely therefore that there are distinct NK cell subsets within the human liver that are not represented in peripheral blood. The biological significance of this for hepatic infections such as HCV is not yet clear as we do not know the function of these tissue resident cells or the relative importance of resident and circulating conventional NK (cNK) cells to the HCV specific immune response. However, information from the murine studies so far suggests that cNK cells that are found in the liver recirculate into the periphery and can function as a read out for hepatic immune responses (Peng et al., 2013). The identification of human counterparts for these trNK cells is of obvious interest for our understanding of the role NK cells play in the immune response to HCV. Finally, the impact of human cytomegalovirus (HCMV) on the NK cell response to HCV, as has previously been defined for hantavirus (Björkström et al., 2011), will also be important to dissect.

In summary, despite all the recent knowledge, it appears that we only understand a fraction of the role that NK cells play in the immune response to pathogen. Specifically in HCV, we need to define what a beneficial NK cell response looks like and if newly defined NK cell lineages also exist in humans. These need to well powered with well-defined control populations. In addition to increasing our basic understanding of how the innate immune system works, further research may open up the opportunity to target NK cells as part of a HCV vaccine strategy, an important research priority within the HCV field.

### Conflict of interest statement

The author declares that the research was conducted in the absence of any commercial or financial relationships that could be construed as a potential conflict of interest.

## References

[B1] Afdhal N. Zeuzem S. Kwo P. Chojkier M. Gitlin N. Puoti M.. (2014). Ledipasvir and sofosbuvir for untreated HCV genotype 1 infection. N. Engl. J. Med. 370, 1889–1898. 10.1056/NEJMoa1402454 24725239

[B2] Ahlenstiel G. Edlich B. Hogdal L. J. Rotman Y. Noureddin M. Feld J. J.. (2011). Early changes in natural killer cell function indicate virologic response to interferon therapy for hepatitis C. Gastroenterology 141, 1231-9, 1239.e1–2. 10.1053/j.gastro.2011.06.069 21741920 PMC3353552

[B3] Ahlenstiel G. Titerence R. H. Koh C. Edlich B. Feld J. J. Rotman Y.. (2010). Natural killer cells are polarized toward cytotoxicity in chronic hepatitis C in an interferon-alfa-dependent manner. Gastroenterology 138, 325-35.e1–2. 10.1053/j.gastro.2009.08.066 19747917 PMC2862622

[B4] Alter G. Jost S. Rihn S. Reyor L. L. Nolan B. E. Ghebremichael M.. (2011). Reduced frequencies of NKp30+NKp46+, CD161+, and NKG2D+ NK cells in acute HCV infection may predict viral clearance. J. Hepatol. 55, 278–288. 10.1016/j.jhep.2010.11.030 21168454 PMC3729214

[B5] Alter H. J. Seeff L. B. (2000). Recovery, persistence, and sequelae in hepatitis C virus infection: a perspective on long-term outcome. Semin. Liver Dis. 20, 17–35. 10.1055/s-2000-9505 10895429

[B6] Amadei B. Urbani S. Cazaly A. Fisicaro P. Zerbini A. Ahmed P.. (2010). Activation of natural killer cells during acute infection with hepatitis C virus. Gastroenterology 138, 1536–1545. 10.1053/j.gastro.2010.01.006 20080094 PMC4183834

[B7] Anfossi N. André P. Guia S. Falk C. S. Roetynck S. Stewart C. A.. (2006). Human NK cell education by inhibitory receptors for MHC class I. Immunity 25, 331–342. 10.1016/j.immuni.2006.06.013 16901727

[B8] Artis D. Spits H. (2015). The biology of innate lymphoid cells. Nature 517, 293–301. 10.1038/nature14189 25592534

[B9] Baume D. M. Robertson M. J. Levine H. Manley T. J. Schow P. W. Ritz J. (1992). Differential responses to interleukin 2 define functionally distinct subsets of human natural killer cells. Eur. J. Immunol. 22, 1–6. 10.1002/eji.1830220102 1370410

[B10] Baumert T. F. Fauvelle C. Chen D. Y. Lauer G. M. (2014). A prophylactic hepatitis C virus vaccine: a distant peak still worth climbing. J. Hepatol. 61, S34–S44. 10.1016/j.jhep.2014.09.009 25443345

[B11] Björkström N. K. Lindgren T. Stoltz M. Fauriat C. Braun M. Evander M.. (2011). Rapid expansion and long-term persistence of elevated NK cell numbers in humans infected with hantavirus. J. Exp. Med. 208, 13–21. 10.1084/jem.20100762 21173105 PMC3023129

[B12] Boisvert J. Kunkel E. J. Campbell J. J. Keeffe E. B. Butcher E. C. Greenberg H. B. (2003). Liver-infiltrating lymphocytes in end-stage hepatitis C virus: subsets, activation status, and chemokine receptor phenotypes. J. Hepatol. 38, 67–75. 10.1016/S0168-8278(02)00328-8 12480562

[B13] Bonorino P. Ramzan M. Camous X. Dufeu-Duchesne T. Thélu M. A. Sturm N.. (2009). Fine characterization of intrahepatic NK cells expressing natural killer receptors in chronic hepatitis B and C. J. Hepatol. 51, 458–467. 10.1016/j.jhep.2009.05.030 19596474

[B14] Braud V. M. Allan D. S. O'Callaghan C. A. Söderström K. D'Andrea A. Ogg G. S.. (1998). HLA-E binds to natural killer cell receptors CD94/NKG2A, B and C. Nature 391, 795–799. 10.1038/35869 9486650

[B15] Burgess S. Partovi N. Yoshida E. M. Erb S. R. Azalgara V. M. Hussaini T. (2015). A review of drug interactions with direct-acting antivirals for hepatitis C: implications for HIV and transplant patients. Ann. Pharmacother. 49, 674–687. 10.1177/1060028015576180 25770114

[B16] Caligiuri M. A. (2008). Human natural killer cells. Blood 112, 461–469. 10.1182/blood-2007-09-077438 18650461 PMC2481557

[B17] Carganico A. Dupke S. Ehret R. Berg T. Baumgarten A. Obermeier M.. (2014). New dolutegravir resistance pattern identified in a patient failing antiretroviral therapy. J. Int. AIDS Soc. 17:19749. 10.7448/IAS.17.4.19749 25397494 PMC4225390

[B18] Cassidy S. A. Cheent K. S. Khakoo S. I. (2014). Effects of Peptide on NK cell-mediated MHC I recognition. Front. Immunol. 5:133. 10.3389/fimmu.2014.00133 24744756 PMC3978238

[B19] Colonna M. (2009). Interleukin-22-producing natural killer cells and lymphoid tissue inducer-like cells in mucosal immunity. Immunity 31, 15–23. 10.1016/j.immuni.2009.06.008 19604490

[B20] Cooper M. A. Elliott J. M. Keyel P. A. Yang L. Carrero J. A. Yokoyama W. M. (2009). Cytokine-induced memory-like natural killer cells. Proc. Natl. Acad. Sci. U.S.A. 106, 1915–1919. 10.1073/pnas.0813192106 19181844 PMC2644138

[B21] Cooper S. Erickson A. L. Adams E. J. Kansopon J. Weiner A. J. Chien D. Y.. (1999). Analysis of a successful immune response against hepatitis C virus. Immunity 10, 439–449. 10.1016/S1074-7613(00)80044-8 10229187

[B22] Cortez V. S. Fuchs A. Cella M. Gilfillan S. Colonna M. (2014). Cutting edge: salivary gland NK cells develop independently of Nfil3 in steady-state. J. Immunol. 192, 4487–4491. 10.4049/jimmunol.1303469 24740507

[B23] Cox A. L. Page K. Bruneau J. Shoukry N. H. Lauer G. M. Kim A. Y.. (2009). Rare birds in North America: acute hepatitis C cohorts. Gastroenterology 136, 26–31. 10.1053/j.gastro.2008.11.049 19059257 PMC4143376

[B24] Deignan T. Curry M. P. Doherty D. G. Golden-Mason L. Volkov Y. Norris S.. (2002). Decrease in hepatic CD56(+) T cells and V alpha 24(+) natural killer T cells in chronic hepatitis C viral infection. J. Hepatol. 37, 101–108. 10.1016/S0168-8278(02)00072-7 12076868

[B25] De Maria A. Bozzano F. Cantoni C. Moretta L. (2011). Revisiting human natural killer cell subset function revealed cytolytic CD56(dim)CD16+ NK cells as rapid producers of abundant IFN-gamma on activation. Proc. Natl. Acad. Sci. U.S.A. 108, 728–732. 10.1073/pnas.1012356108 21187373 PMC3021076

[B26] De Maria A. Fogli M. Mazza S. Basso M. Picciotto A. Costa P.. (2007). Increased natural cytotoxicity receptor expression and relevant IL-10 production in NK cells from chronically infected viremic HCV patients. Eur. J. Immunol. 37, 445–455. 10.1002/eji.200635989 17273991

[B27] Doherty D. G. O'Farrelly C. (2000). Innate and adaptive lymphoid cells in the human liver. Immunol. Rev. 174, 5–20. 10.1034/j.1600-0528.2002.017416.x 10807503

[B28] Dring M. M. Morrison M. H. McSharry B. P. Guinan K. J. Hagan R. O'Farrelly C.. (2011). Innate immune genes synergize to predict increased risk of chronic disease in hepatitis C virus infection. Proc. Natl. Acad. Sci. U.S.A. 108, 5736–5741. 10.1073/pnas.1016358108 21402922 PMC3078345

[B29] Elliott J. M. Yokoyama W. M. (2011). Unifying concepts of MHC-dependent natural killer cell education. Trends Immunol. 32, 364–372. 10.1016/j.it.2011.06.001 21752715 PMC3151350

[B30] Fehniger T. A. Shah M. H. Turner M. J. VanDeusen J. B. Whitman S. P. Cooper M. A.. (1999). Differential cytokine and chemokine gene expression by human NK cells following activation with IL-18 or IL-15 in combination with IL-12: implications for the innate immune response. J. Immunol. 162, 4511–4520. 10201989

[B31] Ferlazzo G. Moretta L. (2014). Dendritic cell editing by natural killer cells. Crit. Rev. Oncog. 19, 67–75. 10.1615/CritRevOncog.2014010827 24941374

[B32] Fernandez N. C. Treiner E. Vance R. E. Jamieson A. M. Lemieux S. Raulet D. H. (2005). A subset of natural killer cells achieves self-tolerance without expressing inhibitory receptors specific for self-MHC molecules. Blood 105, 4416–4423. 10.1182/blood-2004-08-3156 15728129 PMC1895026

[B33] Fugier E. Marche H. Thélu M. A. Macek Jílková Z. Van Campenhout N. Dufeu-Duchesne T.. (2014). Functions of liver natural killer cells are dependent on the severity of liver inflammation and fibrosis in chronic hepatitis C. PLoS ONE 9:e95614. 10.1371/journal.pone.0095614 24759660 PMC3997478

[B34] Gardiner C. M. (2008). Killer cell immunoglobulin-like receptors on NK cells: the how, where and why. Int. J. Immunogenet. 35, 1–8. 10.1111/j.1744-313x.2007.00739.x 18093180

[B35] Ge D. Fellay J. Thompson A. J. Simon J. S. Shianna K. V. Urban T. J.. (2009). Genetic variation in IL28B predicts hepatitis C treatment-induced viral clearance. Nature 461, 399–401. 10.1038/nature08309 19684573

[B36] Glässner A. Eisenhardt M. Kramer B. Korner C. Coenen M. Sauerbruch T.. (2012). NK cells from HCV-infected patients effectively induce apoptosis of activated primary human hepatic stellate cells in a TRAIL-, FasL- and NKG2D-dependent manner. Lab. Invest. 92, 967–977. 10.1038/labinvest.2012.54 22449797

[B37] Golden-Mason L. Hahn Y. S. Strong M. Cheng L. Rosen H. R. (2014). Extracellular HCV-core protein induces an immature regulatory phenotype in NK cells: implications for outcome of acute infection. PLoS ONE 9:e103219. 10.1371/journal.pone.0103219 25076408 PMC4116173

[B38] Golden-Mason L. Madrigal-Estebas L. McGrath E. Conroy M. J. Ryan E. J. Hegarty J. E.. (2008). Altered natural killer cell subset distributions in resolved and persistent hepatitis C virus infection following single source exposure. Gut 57, 1121–1128. 10.1136/gut.2007.130963 18372499

[B39] Gonzalez-Galarza F. F. Christmas S. Middleton D. Jones A. R. (2011). Allele frequency net: a database and online repository for immune gene frequencies in worldwide populations. Nucleic Acids Res. 39, D913–D919. 10.1093/nar/gkq1128 21062830 PMC3013710

[B40] Hata K. Zhang X. R. Iwatsuki S. Van Thiel D. H. Herberman R. B. Whiteside T. L. (1990). Isolation, phenotyping, and functional analysis of lymphocytes from human liver. Clin. Immunol. Immunopathol. 56, 401–419. 10.1016/0090-1229(90)90160-R 1697226

[B41] Hedskog C. Dvory-Sobol H. Gontcharova V. Martin R. Ouyang W. Han B.. (2015). Evolution of the HCV viral population from a patient with S282T detected at relapse after sofosbuvir monotherapy. J. Viral Hepat. [Epub ahead of print]. 10.1111/jvh.12405 25784085

[B42] Herzer K. Falk C. S. Encke J. Eichhorst S. T. Ulsenheimer A. Seliger B.. (2003). Upregulation of major histocompatibility complex class I on liver cells by hepatitis C virus core protein via p53 and TAP1 impairs natural killer cell cytotoxicity. J. Virol. 77, 8299–8309. 10.1128/JVI.77.15.8299-8309.2003 12857899 PMC165225

[B43] Horowitz A. Behrens R. H. Okell L. Fooks A. R. Riley E. M. (2010). NK cells as effectors of acquired immune responses: effector CD4+ T cell-dependent activation of NK cells following vaccination. J. Immunol. 185, 2808–2818. 10.4049/jimmunol.1000844 20679529

[B44] Jacobs R. Hintzen G. Kemper A. Beul K. Kempf S. Behrens G.. (2001). CD56bright cells differ in their KIR repertoire and cytotoxic features from CD56dim NK cells. Eur. J. Immunol. 31, 3121–3127. 10.1002/1521-4141(2001010)31:10<3121::AID-IMMU3121>3.0.CO;2-4 11592089

[B45] Jang J. Y. Kim S. J. Cho E. K. Jeong S. W. Park E. J. Lee W. C.. (2014). TRAIL enhances apoptosis of human hepatocellular carcinoma cells sensitized by hepatitis C virus infection: therapeutic implications. PLoS ONE 9:e98171. 10.1371/journal.pone.0098171 24927176 PMC4057066

[B46] Ji H. Kozak R. A. Biondi M. J. Pilon R. Vallee D. Liang B. B.. (2015). Next generation sequencing of the hepatitis C virus NS5B gene reveals potential novel S282 drug resistance mutations. Virology 477, 1–9. 10.1016/j.virol.2014.12.037 25600207

[B47] Jinushi M. Takehara T. Tatsumi T. Kanto T. Miyagi T. Suzuki T.. (2004). Negative regulation of NK cell activities by inhibitory receptor CD94/NKG2A leads to altered NK cell-induced modulation of dendritic cell functions in chronic hepatitis C virus infection. J. Immunol. 173, 6072–6081. 10.4049/jimmunol.173.10.6072 15528343

[B48] Joncker N. T. Fernandez N. C. Treiner E. Vivier E. Raulet D. H. (2009). NK cell responsiveness is tuned commensurate with the number of inhibitory receptors for self-MHC class I: the rheostat model. J. Immunol. 182, 4572–4580. 10.4049/jimmunol.0803900 19342631 PMC2938179

[B49] Kawarabayashi N. Seki S. Hatsuse K. Ohkawa T. Koike Y. Aihara T.. (2000). Decrease of CD56(+)T cells and natural killer cells in cirrhotic livers with hepatitis C may be involved in their susceptibility to hepatocellular carcinoma. Hepatology 32, 962–969. 10.1053/jhep.2000.19362 11050046 PMC7165992

[B50] Keane C. O'shea D. Reiberger T. Peck-Radosavljevic M. Farrell G. Bergin C.. (2013). Variation in both IL28B and KIR2DS3 genes influence pegylated interferon and ribavirin hepatitis C treatment outcome in HIV-1 co-infection. PLoS ONE 8:e66831. 10.1371/journal.pone.0066831 23826153 PMC3691248

[B51] Khakoo S. I. Thio C. L. Martin M. P. Brooks C. R. Gao X. Astemborski J.. (2004). HLA and NK cell inhibitory receptor genes in resolving hepatitis C virus infection. Science 305, 872–874. 10.1126/science.1097670 15297676

[B52] Kim S. Poursine-Laurent J. Truscott S. M. Lybarger L. Song Y. J. Yang L.. (2005). Licensing of natural killer cells by host major histocompatibility complex class I molecules. Nature 436, 709–713. 10.1038/nature03847 16079848

[B53] Knapp S. Warshow U. Hegazy D. Brackenbury L. Guha I. N. Fowell A.. (2010). Consistent beneficial effects of killer cell immunoglobulin-like receptor 2DL3 and group 1 human leukocyte antigen-C following exposure to hepatitis C virus. Hepatology 51, 1168–1175. 10.1002/hep.23477 20077564 PMC4202114

[B54] Kokordelis P. Krämer B. Körner C. Boesecke C. Voigt E. Ingiliz P.. (2014). An effective interferon-gamma-mediated inhibition of hepatitis C virus replication by natural killer cells is associated with spontaneous clearance of acute hepatitis C in human immunodeficiency virus-positive patients. Hepatology 59, 814–827. 10.1002/hep.26782 24382664

[B55] Larkin J. Bost A. Glass J. I. Tan S. L. (2006). Cytokine-activated natural killer cells exert direct killing of hepatoma cells harboring hepatitis C virus replicons. J. interferon Cytokine Res. 26, 854–865. 10.1089/jir.2006.26.854 17238828

[B56] Lechner F. Wong D. K. Dunbar P. R. Chapman R. Chung R. T. Dohrenwend P.. (2000). Analysis of successful immune responses in persons infected with hepatitis C virus. J. Exp. Med. 191, 1499–1512. 10.1084/jem.191.9.1499 10790425 PMC2213430

[B57] Lee N. Llano M. Carretero M. Ishitani A. Navarro F. López-Botet M.. (1998). HLA-E is a major ligand for the natural killer inhibitory receptor CD94/NKG2A. Proc. Natl. Acad. Sci. U.S.A. 95, 5199–5204. 10.1073/pnas.95.9.5199 9560253 PMC20238

[B58] Lohmann V. Bartenschlager R. (2014). On the history of hepatitis C virus cell culture systems. J. Med. Chem. 57, 1627–1642. 10.1021/jm401401n 24164647

[B59] Micallef J. M. Kaldor J. M. Dore G. J. (2006). Spontaneous viral clearance following acute hepatitis C infection: a systematic review of longitudinal studies. J. Viral Hepat. 13, 34–41. 10.1111/j.1365-2893.2005.00651.x 16364080

[B60] Moesta A. K. Graef T. Abi-Rached L. Older Aguilar A. M. Guethlein L. A. Parham P. (2010). Humans differ from other hominids in lacking an activating NK cell receptor that recognizes the C1 epitope of MHC class I. J. Immunol. 185, 4233–4237. 10.4049/jimmunol.1001951 20802150 PMC3124310

[B61] Moesta A. K. Norman P. J. Yawata M. Yawata N. Gleimer M. Parham P. (2008). Synergistic polymorphism at two positions distal to the ligand-binding site makes KIR2DL2 a stronger receptor for HLA-C than KIR2DL3. J. Immunol. 180, 3969–3979. 10.4049/jimmunol.180.6.3969 18322206

[B62] Montes-Cano M. A. Caro-Oleas J. L. Romero-Gómez M. Diago M. Andrade R. Carmona I.. (2005). HLA-C and KIR genes in hepatitis C virus infection. Hum. Immunol. 66, 1106–1109. 10.1016/j.humimm.2006.02.001 16571411

[B63] Morishima C. Paschal D. M. Wang C. C. Yoshihara C. S. Wood B. L. Yeo A. E.. (2006). Decreased NK cell frequency in chronic hepatitis C does not affect *ex vivo* cytolytic killing. Hepatology 43, 573–580. 10.1002/hep.21073 16496327

[B64] Narni-Mancinelli E. Jaeger B. N. Bernat C. Fenis A. Kung S. De Gassart A.. (2012). Tuning of natural killer cell reactivity by NKp46 and Helios calibrates T cell responses. Science 335, 344–348. 10.1126/science.1215621 22267813

[B65] Nattermann J. (2011). NK cells in acute hepatitis C. J. Hepatol. 55, 265–267. 10.1016/j.jhep.2011.01.005 21236311

[B66] Nattermann J. Nischalke H. D. Hofmeister V. Ahlenstiel G. Zimmermann H. Leifeld L.. (2005). The HLA-A2 restricted T cell epitope HCV core 35-44 stabilizes HLA-E expression and inhibits cytolysis mediated by natural killer cells. Am. J. Pathol. 166, 443–453. 10.1016/S0002-9440(10)62267-5 15681828 PMC1602324

[B67] Norris S. Doherty D. G. Collins C. McEntee G. Traynor O. Hegarty J. E.. (1999). Natural T cells in the human liver: cytotoxic lymphocytes with dual T cell and natural killer cell phenotype and function are phenotypically heterogenous and include Valpha24-JalphaQ and gammadelta T cell receptor bearing cells. Hum. Immunol. 60, 20–31. 10.1016/S0198-8859(98)00098-6 9952024

[B68] O'Connor G. M. Guinan K. J. Cunningham R. T. Middleton D. Parham P. Gardiner C. M. (2007). Functional polymorphism of the KIR3DL1/S1 receptor on human NK cells. J. Immunol. 178, 235–241. 10.4049/jimmunol.178.1.235 17182560

[B69] Oliviero B. Varchetta S. Paudice E. Michelone G. Zaramella M. Mavilio D.. (2009). Natural killer cell functional dichotomy in chronic hepatitis B and chronic hepatitis C virus infections. Gastroenterology 137, 1151-60, 1160.e1–7. 10.1053/j.gastro.2009.05.047 19470388

[B70] Parham P. Norman P. J. Abi-Rached L. Guethlein L. A. (2012). Human-specific evolution of killer cell immunoglobulin-like receptor recognition of major histocompatibility complex class I molecules. Philos. Trans. R. Soc. Lond. B. Biol. Sci. 367, 800–811. 10.1098/rstb.2011.0266 22312047 PMC3267113

[B71] Park S. H. Rehermann B. (2014). Immune responses to HCV and other hepatitis viruses. Immunity 40, 13–24. 10.1016/j.immuni.2013.12.010 24439265 PMC4480226

[B72] Pelletier S. Drouin C. Bédard N. Khakoo S. I. Bruneau J. Shoukry N. H. (2010). Increased degranulation of natural killer cells during acute HCV correlates with the magnitude of virus-specific T cell responses. J. Hepatol. 53, 805–816. 10.1016/j.jhep.2010.05.013 20688412 PMC4178223

[B73] Peng H. Jiang X. Chen Y. Sojka D. K. Wei H. Gao X.. (2013). Liver-resident NK cells confer adaptive immunity in skin-contact inflammation. J. Clin. Invest. 123, 1444–1456. 10.1172/JCI66381 23524967 PMC3613925

[B74] Peppa D. Gill U. S. Reynolds G. Easom N. J. Pallett L. J. Schurich A.. (2013). Up-regulation of a death receptor renders antiviral T cells susceptible to NK cell-mediated deletion. J. Exp. Med. 210, 99–114. 10.1084/jem.20121172 23254287 PMC3549717

[B75] Prokunina-Olsson L. Muchmore B. Tang W. Pfeiffer R. M. Park H. Dickensheets H.. (2013). A variant upstream of IFNL3 (IL28B) creating a new interferon gene IFNL4 is associated with impaired clearance of hepatitis C virus. Nat. Genet. 45, 164–171. 10.1038/ng.2521 23291588 PMC3793390

[B76] Rajagopalan S. Long E. O. (1997). The direct binding of a p58 killer cell inhibitory receptor to human histocompatibility leukocyte antigen (HLA)-Cw4 exhibits peptide selectivity. J. Exp. Med. 185, 1523–1528. 10.1084/jem.185.8.1523 9126935 PMC2196286

[B77] Rauch A. Laird R. McKinnon E. Telenti A. Furrer H. Weber R. (2007). Influence of inhibitory killer immunoglobulin-like receptors and their HLA-C ligands on resolving hepatitis C virus infection. Tissue Antigens 69(Suppl. 1), 237–240. 10.1111/j.1399-0039.2006.773_4.x 17445209

[B78] Rehermann B. (2009). Hepatitis C virus versus innate and adaptive immune responses: a tale of coevolution and coexistence. J. Clin. Invest. 119, 1745–1754. 10.1172/JCI39133 19587449 PMC2701885

[B79] Satoh M. Seki S. Hashimoto W. Ogasawara K. Kobayashi T. Kumagai K.. (1996). Cytotoxic gammadelta or alphabeta T cells with a natural killer cell marker, CD56, induced from human peripheral blood lymphocytes by a combination of IL-12 and IL-2. J. Immunol. 157, 3886–3892. 8892619

[B80] Séne D. Levasseur F. Abel M. Lambert M. Camous X. Hernandez C.. (2010). Hepatitis C virus (HCV) evades NKG2D-dependent NK cell responses through NS5A-mediated imbalance of inflammatory cytokines. PLoS Pathog. 6:e1001184. 10.1371/journal.ppat.1001184 21085608 PMC2978723

[B81] Serti E. Chepa-Lotrea X. Kim Y. J. Keane M. Fryzek N. Liang T. J.. (2015). Successful interferon-free therapy of chronic hepatitis C virus infection normalizes natural killer cell function. Gastroenterology 149, 190–200. 10.1053/j.gastro.2015.03.004 25754160 PMC4523392

[B82] Shepard C. W. Finelli L. Alte M. J. (2005). Global epidemiology of hepatitis C virus infection. Lancet. Infect. Dis. 5, 558–567. 10.1016/S1473-3099(05)70216-4 16122679

[B83] Soderquest K. Walzer T. Zafirova B. Klavinskis L. S. Polic B. Vivier E.. (2011). Cutting edge: CD8+ T cell priming in the absence of NK cells leads to enhanced memory responses. J. Immunol. 186, 3304–3308. 10.4049/jimmunol.1004122 21307295

[B84] Sojka D. K. Plougastel-Douglas B. Yang L. Pak-Wittel M. A. Artyomov M. N. Ivanova Y.. (2014). Tissue-resident natural killer (NK) cells are cell lineages distinct from thymic and conventional splenic NK cells. Elife 3:e01659. 10.7554/eLife.01659 24714492 PMC3975579

[B85] Soriano V. Labarga P. Barreiro P. Fernandez-Montero J. V. de Mendoza C. Esposito I.. (2015). Drug interactions with new hepatitis C oral drugs. Expert Opin. Drug Metab. Toxicol. 11, 333–341. 10.1517/17425255.2015.998997 25553890

[B86] Stegmann K. A. Björkström N. K. Veber H. Ciesek S. Riese P. Wiegand J.. (2010). Interferon-alpha-induced TRAIL on natural killer cells is associated with control of hepatitis C virus infection. Gastroenterology 138, 1885–1897. 10.1053/j.gastro.2010.01.051 20334827

[B87] Sun J. C. Beilke J. N. Lanier L. L. (2009). Adaptive immune features of natural killer cells. Nature 457, 557–561. 10.1038/nature07665 19136945 PMC2674434

[B88] Suppiah V. Moldovan M. Ahlenstiel G. Berg T. Weltman M. Abate M. L.. (2009). IL28B is associated with response to chronic hepatitis C interferon-alpha and ribavirin therapy. Nat. Genet. 41, 1100–1104. 10.1038/ng.447 19749758

[B89] Tanaka Y. Nishida N. Sugiyama M. Kurosaki M. Matsuura K. Sakamoto N.. (2009). Genome-wide association of IL28B with response to pegylated interferon-alpha and ribavirin therapy for chronic hepatitis C. Nat. Genet. 41, 1105–1109. 10.1038/ng.449 19749757

[B90] Thimme R. Bukh J. Spangenberg H. C. Wieland S. Pemberton J. Steiger C.. (2002). Viral and immunological determinants of hepatitis C virus clearance, persistence, and disease. Proc. Natl. Acad. Sci. U.S.A. 99, 15661–15668. 10.1073/pnas.202608299 12441397 PMC137773

[B91] Thoens C. Berger C. Trippler M. Siemann H. Lutterbeck M. Broering R.. (2014). KIR2DL3(+)NKG2A(-) natural killer cells are associated with protection from productive hepatitis C virus infection in people who inject drugs. J. Hepatol. 61, 475–481. 10.1016/j.jhep.2014.04.020 24780303

[B92] Thomas D. L. Thio C. L. Martin M. P. Qi Y. Ge D. O'Huigin C.. (2009). Genetic variation in IL28B and spontaneous clearance of hepatitis C virus. Nature 461, 798–801. 10.1038/nature08463 19759533 PMC3172006

[B93] Tillmann H. L. Thompson A. J. Patel K. Wiese M. Tenckhoff H. Nischalke H. D.. (2010). A polymorphism near IL28B is associated with spontaneous clearance of acute hepatitis C virus and jaundice. Gastroenterology 139, 1586–1592.e11. 10.1053/j.gastro.2010.07.005 20637200

[B94] VandenBussche C. J. Mulrooney T. J. Frazier W. R. Dakshanamurthy S. Hurley C. K. (2009). Dramatically reduced surface expression of NK cell receptor KIR2DS3 is attributed to multiple residues throughout the molecule. Genes Immun. 10, 162–173. 10.1038/gene.2008.91 19005473 PMC3487464

[B95] Varchetta S. Mele D. Mantovani S. Oliviero B. Cremonesi E. Ludovisi S.. (2012). Impaired intrahepatic natural killer cell cytotoxic function in chronic hepatitis C virus infection. Hepatology 56, 841–849. 10.1002/hep.25723 22431186

[B96] Vercauteren K. de Jong Y. P. Meuleman P. (2014). HCV animal models and liver disease. J. Hepatol. 61, S26–S33. 10.1016/j.jhep.2014.07.013 25443343

[B97] Vilches C. Parham P. (2002). KIR: diverse, rapidly evolving receptors of innate and adaptive immunity. Annu. Rev. Immunol. 20, 217–251. 10.1146/annurev.immunol.20.092501.134942 11861603

[B98] Waggoner S. N. Cornberg M. Selin L. K. Welsh R. M. (2012). Natural killer cells act as rheostats modulating antiviral T cells. Nature 481, 394–398. 10.1038/nature10624 22101430 PMC3539796

[B99] Wang S. H. Huang C. X. Ye L. Wang X. Song L. Wang Y. J.. (2008). Natural killer cells suppress full cycle HCV infection of human hepatocytes. J. Viral Hepat. 15, 855–864. 10.1111/j.1365-2893.2008.01014.x 18637071 PMC2675875

[B100] Werner J. M. Heller T. Gordon A. M. Sheets A. Sherker A. H. Kessler E. (2013). Innate immune responses in hepatitis C virus-exposed healthcare workers who do not develop acute infection. Hepatology 58, 1621–1631. 10.1002/hep.26353 23463364 PMC3688637

[B101] Zeuzem S. Andreone P. Pol S. Lawitz E. Diago M. Roberts S.. (2011). Telaprevir for retreatment of HCV infection. N. Engl. J. Med. 364, 2417–2428. 10.1056/NEJMoa1013086 21696308

[B102] Zeuzem S. Dusheiko G. M. Salupere R. Mangia A. Flisiak R. Hyland R. H.. (2014). Sofosbuvir and ribavirin in HCV genotypes 2 and 3. N. Engl. J. Med. 370, 1993–2001. 10.1056/NEJMoa1316145 24795201

